# Enhancing fibroblast–epithelial cell communications: Serpine2 as a key molecule in *Fusobacterium nucleatum*–promoted colon cancer

**DOI:** 10.3389/fimmu.2025.1563922

**Published:** 2025-06-26

**Authors:** Xueke Li, Simin Luo, Yifang Jiang, Qiong Ma, Fengming You, Qixuan Kuang, Xi Fu, Chuan Zheng

**Affiliations:** ^1^ Traditional Chinese Medicine (TCM) Regulating Metabolic Diseases Key Laboratory of Sichuan Province, Hospital of Chengdu University of Traditional Chinese Medicine, Chengdu, Sichuan, China; ^2^ Oncology Teaching and Research Department, Chengdu University of Traditional Chinese Medicine, Chengdu, Sichuan, China; ^3^ Institute of Oncology, Chengdu University of Traditional Chinese Medicine, Chengdu, Sichuan, China

**Keywords:** single cell RNA-seq, *Fusobacterium nucleatum*, colon cancer, SerpinE2, fibroblast

## Abstract

**Background:**

*Fusobacterium nucleatum* (*Fn*) has been identified as a causative factor in the progression of colon cancer. This study aims to integrate bulk RNA-seq with single-cell RNA-seq (scRNA-seq) to elucidate the molecular mechanisms by which *Fn* facilitates colon cancer progression.

**Methods:**

The scRNA-seq data from tumor tissues of *Fn* intervention were analyzed to screen cells with significant proportion changes. Differentially expressed genes of cells with different proportions were extracted and intersected with those identified in the bulk RNA-seq analysis. Three machine learning algorithms were employed to identify characteristic genes. Clinical tissue samples and external datasets, along with *in vitro* co-culture experiments, were utilized to validate these findings.

**Results:**

Following *Fn* intervention, there was an observed increase in the fibroblast iso-cellular ratio and interaction levels. Utilizing machine learning algorithms, we identified five key genes. The differential expression of Serpine2 was validated using clinical samples and external datasets. Furthermore, patients with metastatic colon cancer exhibited significantly higher Serpine2 expression compared to those without metastasis. *Fn* was found to significantly enhance the expression of Serpine2 in fibroblasts and to promote the proliferation and migration capabilities of tumor cells.

**Conclusion:**

This study elucidates the role of *Fn* in promoting colon cancer progression through the enhancement of fibro-macrophage–epithelial cell interactions. Furthermore, Serpine2 has been identified as a potential molecular marker associated with *Fn*-mediated colon cancer progression and metastasis. These findings contribute novel insights that may inform the development of therapeutic strategies for colon cancer.

## 1 Introduction

Colon cancer is one of the most common tumors worldwide, and its incidence is at the forefront of major malignancies worldwide. In 2022, microbial polymorphisms have emerged as a novel characteristic of tumors (1), with significant implications for the prevention, diagnosis, and treatment of colon cancer (2). Among them, *Fusobacterium nucleatum* (*Fn*), a microorganism found in both the oral cavity and the intestinal tract, has garnered considerable attention for its cancer-promoting effects on colon cancer (3, 4). However, in colorectal cancer tumors, this bacterium is abnormally abundant and is strongly associated with cancer recurrence, metastasis, and poor patient prognosis (5–7). *Fn* is able to attach to tumor tissues through various adhesion proteins (such as FadA (8), Fap2 (9), and RadD (10)) and stimulate tumor formation, thereby accelerating disease progression. Furthermore, *Fn* has been reported to have a strong association with the level of CD8^+^ T-cell infiltration within the tumor microenvironment, which subsequently influences the efficacy of immunotherapy for colon cancer (11).

Although numerous studies have investigated the mechanisms by which *Fn* facilitates colon cancer progression, to the best of our knowledge, no research has yet integrated bulk RNA sequencing with single-cell RNA sequencing (scRNA-seq) to explore the progression of *Fn*-promoted colon cancer. For example, a study analyzed the tumor microenvironment of colon cancer mice (with or without *Fn* infection) treated with anti-PD-1 by scRNA-seq (12). There are also studies identifying *Fn*-promoted PD-L1 expression in colon tumor cells by m6A-seq and RNA-seq (13). Because bulk RNA-seq provides a comprehensive and macroscopic representation of gene expression in the overall tissue, it provides us with a broad outline of gene expression within the tumor tissue. In contrast, scRNA-seq captures gene expression differences at the individual cell level, which helps us to explore the complex and subtle changes in the tumor microenvironment (e.g., tumor cells versus surrounding immune cells and stromal cells) in a more in-depth and detailed manner. Therefore, we investigated the molecular mechanisms through which *Fn* facilitates the progression of colon cancer by integrating bulk RNA-seq and scRNA-seq data from public databases. Our analysis identified Serpine2 as a key factor associated with the enhancement of fibroblast–epithelial cell interactions, contributing to colon cancer progression and metastasis. While previous studies have reported elevated levels of Serpine2 in colon adenocarcinoma, the specific molecular mechanisms by which Serpine2 promotes colon cancer progression at the scRNA-seq level remain unexplored (14). Furthermore, there is a notable gap in understanding how Serpine2 expression changes within the tumor microenvironment following *Fn* infection, which may drive colon cancer progression. Our findings provide valuable insights into the role of Serpine2 in *Fn*-mediated colon cancer progression.

**Figure 1:**
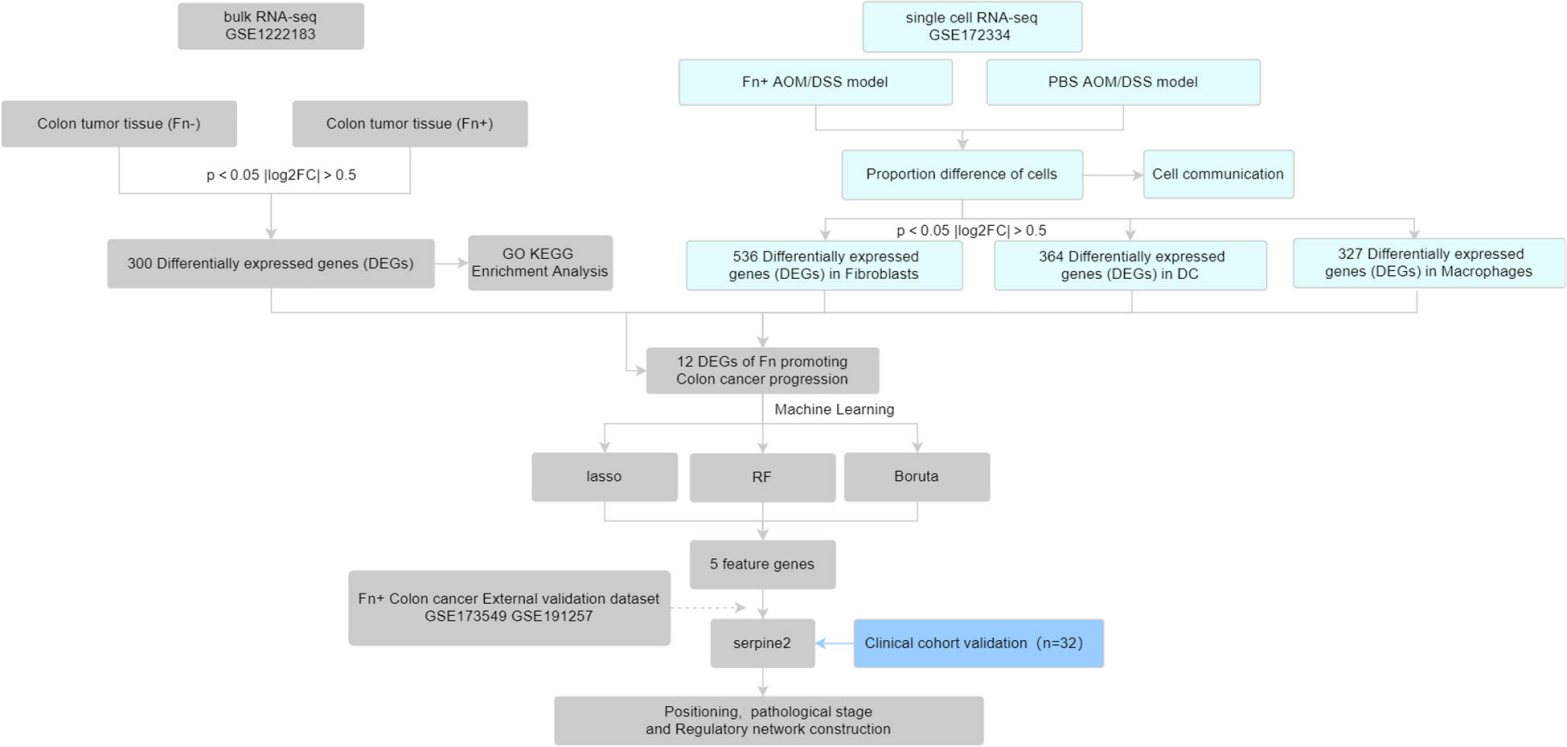
The work roadmap.

In this study, we first obtained *Fn*+ and *Fn*− colon cancer tumor tissue sequencing data from the Gene Expression Omnibus (GEO) database, which were downloaded for differential analysis. Subsequently, we combined single-cell sequencing data from colon cancer mice [*Fn* or phosphate-buffered saline (PBS) intervention] to screen for *Fn*-regulated colon cancer differential genes. The obtained differential genes were further screened for signature genes using machine learning algorithms and validated with external datasets and collected clinical samples. See the work roadmap (**Figure 1**) for details.

## 2 Materials and methods

### 2.1 Data set download

Tumor tissue bulk RNA-seq data (GSE122183) from colon cancer patients (containing *Fn*-positive/negative colon cancer) and colon tumor tissue scRNA-seq (GSE172334) from the Azoxymethane /Dextran Sodium Sulfate (AOM/DSS) colon cancer animal model (*Fn* or PBS intervention) were downloaded from the GEO database as the analyzed dataset. In addition, RNA-seq of *Fn* co-cultured colon cancer cell lines LoVo (GSE173549) and HT29 (GSE191257) were downloaded as validation datasets to independently validate the results of the analyzed datasets. In these datasets, the HT29 cell line was infected *Fn* [multiplicity of infection (MOI) of 300] and either *E. coli* (MOI of 300) or heat-inactivated *Fn* co-culture as a control for 96 h. The LoVo cell line was infected with *Fn* (MOI of 100), and either PBS or *E. coli* (MOI of 50) was used as a control for 24 h.

### 2.2 Identification of differentially expressed genes in *Fn*+ colon cancer

Differential analysis of bulk RNA-seq of colon cancer tumor tissues of *Fn*+ and *Fn*− was performed using the limma package, and P < 0.05 and |log2FC| > 0.5 were set as the conditions to screen differentially expressed genes (DEGs), where P < 0.05 and log2FC > 0.5 for upregulated DEGs and P < 0.05 and log2FC < −0.5 for downregulated DEGs. The differential gene clusters of *Fn* promoting colon cancer development were screened. The volcano map and heat map were drawn to show the results of differential analysis.

### 2.3 Enrichment analysis

Enrichment analysis of DEGs was conducted using the clusterProfiler package in conjunction with the DAVID database. Gene Ontology (GO) and Kyoto Encyclopedia of Genes and Genomes (KEGG) pathway enrichment analyses were performed to explore the phenotypes and pathways associated with the differential genes. The enrichment analysis results of the top five pathways are shown in order of p-value from smallest to largest, respectively.

### 2.4 Single-cell analysis

Seurat package was used to extract 10× single-cell sequencing data. Before Quality Control (QC), different samples were observed for significant batch effects, and outliers were manually eliminated by filtering low-quality cells/genes and after downscaling. The data were normalized after removing mitochondrial genes with expression greater than 20% and genes with expression less than 200. After principal components analysis downscaling, the appropriate number of clusters was filtered by JackStraw and ElbowPlot methods, and the Uniform Manifold Approximation and Projection (umap) clustering map was drawn. Individual clusters were annotated using singleR package with celldex package. The annotated cell clusters were divided into PBS group and *Fn* group, and the difference analysis was also performed to obtain the cell clusters with significant changes in cell proportions. The clusters were extracted for pseudo-bulk RNA-seq difference analysis to extract the DEGs in the differently proportioned cells. The annotated cells were analyzed for communication using the cellchat package to assess the number and strength of intercellular interactions.

### 2.5 Machine learning screening of characterized genes

The differential genes obtained by GSE172334 scRNA-seq were intersected with the DEGs obtained by GSE122183 bulk RNA-seq to take the intersection of the DEGs, and the differential genes of *Fn* promoting colon cancer metastasis were obtained. Three machine learning methods were used to screen feature genes for this differential gene cluster obtained: glmnet package was used to perform lasso regression analysis; after inputting the gene expression matrix, the number of random seeds was set; and 10-fold cross-validation was set to train the model to screen the feature genes. The Boruta package was used for Boruta feature variable screening. The randomForest package was used to construct a random forest model to screen important variables. Finally, the obtained feature genes were taken as intersection to obtain the key genes of *Fn* promoting colon cancer.

### 2.6 Expression validation

Validation was carried out using downloaded external datasets to draw histograms and assess whether there is any difference in the expression of the key genes. The key genes that can robustly pass the validation of different datasets are screened.

### 2.7 Localization and functional analysis

The localization and expression levels of key genes on cell clusters were demonstrated using the Seurat package. GEPIA2 (http://gepia2.cancer-pku.cn/#index) was used to plot Kaplan–Meier (KM) survival curves of key genes to assess their prognostic effects. The TISIDB (http://cis.hku.hk/TISIDB/index.php) was used to assess the use of the NetworkAnalyst database (https://www.networkanalyst.ca/NetworkAnalyst/) to predict the related transcription factors of the key genes with the microRNAs (miRNAs) to map the Transcription factor (TF)-gene-miRNA regulatory network.

### 2.8 Participant recruitment

A cohort of 32 Chinese individuals was recruited to undergo a standard colonoscopy procedure at the Hospital of Chengdu University of Traditional Chinese Medicine in Chengdu, China. Informed written consent was obtained from all participants prior to their inclusion in the study. The study protocol received approval from the ethics committee of the Hospital of Chengdu University of Traditional Chinese Medicine (approval number: 2023KL-096). An overview of the participants’ demographic and clinical characteristics is provided in **Supplementary Table 1**.

### 2.9 Clinical sample collection

Two different anatomical locations were sampled from colon cancer patients during surgeries (15): 1) primary tumor tissues, and 2) normal mucosas (usually 10–20 cm from the tumor, close to the surgical resection borders). After surgical excision, the main tumor and normal tissues were removed within 30 min. They were then immediately moved to liquid nitrogen, chopped into pieces on dry ice, and then preserved at −80°C for further tests.

### 2.10 Real-time fluorescence quantitative polymerase chain reaction

The mRNA expression levels of key genes in clinical samples were evaluated using real-time fluorescence quantitative polymerase chain reaction (PCR) on the tumor and normal tissues of patients with colon cancer. Total tissue RNA was extracted and reverse-transcribed into cDNA for real-time fluorescence quantitative PCR. PCR primers were as follows: human Serpine2: AATGAAACCAGGGATATGATTGAC (forward) and TTGCAAGATATGAGAAACATGGAG (backward); human JunB Proto-Oncogene (JUNB): ACGACTCATACACAGCTACGG (forward) and GCTCGGGTTTCAGGAGTTTGTAGT (backward); human Latent Transforming Growth Factor Beta Binding Protein 4 (LTBP4): CTGCCCATTCTGCGGAACAT (forward) and GCCGAGTAGTGGTAACCAGG (backward); human Matrix Metallopeptidase 7 (MMP7): CATGATTGGCTTTGCGCGAG (forward) and AGACTGCTACCATCCGTCCA (backward); and human β-actin: GTCCACCGCAAATGCTTCTA (forward) and TGCTGTCACCTTCACCGTTC (backward).

### 2.11 Western blot

Total protein was lysed and extracted using the kit. Following bicinchoninic acid quantification, immunoblot analysis was carried out to determine the levels of protein expression of important genes in the clinical samples. hSerpinE2 antibody: cat no. 82732-5-RR, Wuhan Three Eagles. GAPDH antibody: cat no. ab181602, Abcam.

### 2.12 Cell culture

Human colon cancer cells (HCT116, serial: TCHu99) and human colon fibroblasts (CDD-18Co cells, serial: GNHu59) were procured from the cell bank of the National Collection of Authenticated Cell Cultures. The HCT116 cells were maintained in a medium comprising 89% Dulbecco's Modified Eagle Medium (DMEM), 10% Fetal Bovine Serum (FBS), and 1% double antibiotic and were cultured until reaching the logarithmic phase. Similarly, CDD-18Co cells were cultured in a medium containing 89% DMEM, 10% FBS, 1% dual antibiotic, Tumor necrosis factor α (TNF-α) (6 ng/mL), and Transforming growth factor β (TGF-β) (10 ng/mL) and were also cultured to the logarithmic phase for subsequent use. Both cell lines were incubated at 37°C in an atmosphere of 5% CO_2_.

### 2.13 Bacterial culture

The bacterial culture of *Fn* (ATCC 25586) was procured from the Guangdong Microbial Culture Collection Center. Following recovery on a blood agar plate, *Fn* was inoculated into brain heart infusion broth (16). Upon reaching the logarithmic growth phase, the bacterial suspension was centrifuged at 3,000 rpm for 2 min. The supernatant was discarded, and the pellet was resuspended in PBS. The bacterial concentration was then adjusted to 1 × 10^8^ Colony Forming Unit (CFU)/mL and 2 × 10^8^ CFU/mL for subsequent use.

### 2.14 Fibroblast co-culture with *Fn*

For the co-culture experiments with fibroblasts, CDD-18Co cells in the logarithmic phase were seeded into six-well plates at a density of 1 × 10^5^ cells per well. Once the cells adhered to the plate, 100 µL of *Fn* bacterial suspension at MOIs of 100 and 200 was added to the wells to establish co-cultures (17). After 6 h of co-culture, the medium was replaced, and the cells were incubated for an additional 30 h. The supernatant was then centrifuged and filtered for use in the culture of HCT116 cells. CDD-18Co cells remaining in the original wells were subjected to trypsinization to facilitate cell collection for Western blot.

### 2.15 CCK-8 assay

HCT116 cells in the logarithmic growth phase were seeded into 96-well plates at a density of 0.05 × 10^5^ cells per well. Following cell adhesion, the culture medium was replaced with either the supernatant from fibroblast cultures not co-cultured with *Fn* or the supernatant from *Fn*-fibroblast co-cultures, at MOIs of 100 and 200, respectively. After incubation periods of 24 h and 48 h, 10 µL of Cell Counting Kit 8 (CCK-8) reagent was added to each well. The absorbance at 450 nm was subsequently measured using a Thermo Fisher enzyme labeler following a 2-h incubation at 37°C.

### 2.16 Scratch experiment

HCT116 cells in the logarithmic phase were seeded into 12-well plates at a density of 0.5 × 10^5^ cells per well. Once the cells adhered, scratches were introduced using a sterile pipette tip. The wells were washed with PBS to remove cell debris, and the supernatant from the *Fn*-fibroblast co-culture medium was added. Scratch closure was monitored at 24 h and 48 h post-treatment. Images were captured, and the scratched area was quantified using ImageJ software. The percentage of scratch closure was calculated relative to the initial scratched area.

## 3 Results

### 3.1 Differentially expressed genes are closely related to metal ion signaling

**Figure 2:**
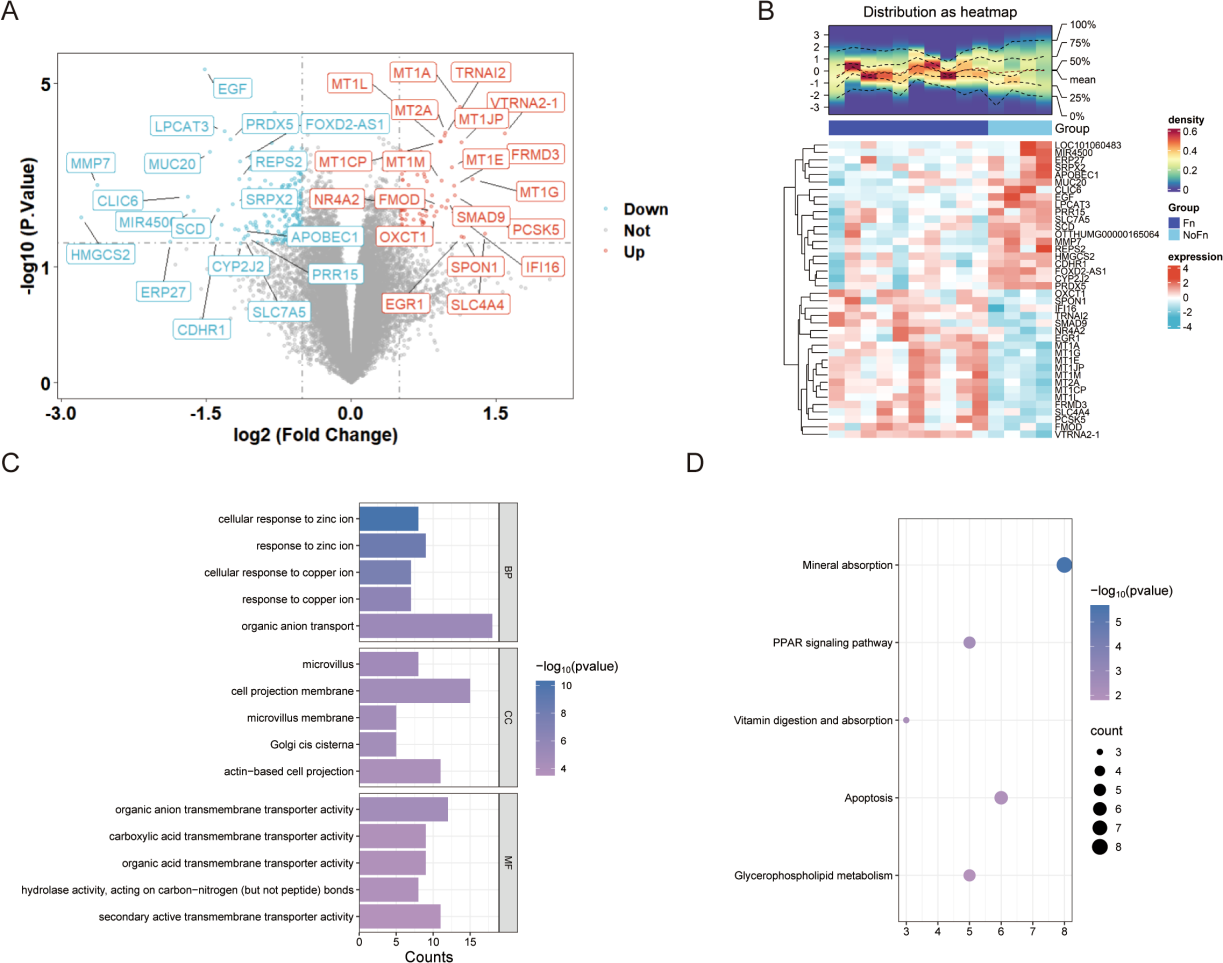
*Fn* promotes colon cancer progression/metastasis, which is closely related to metal ion signaling (GSE122183). **(A)** Difference analysis volcano plot. **(B)** Clustering heat map. **(C)** GO enrichment analysis histogram. **(D)** KEGG enrichment analysis bubble plot.

A total of 300 DEGs (**Figure 2A**, 100 upregulated and 200 downregulated genes) were obtained by limma package difference analysis. The clustering heat map demonstrated the expression levels of these DEGs between the two groups, and we noticed a significant increase in the expression of genes of the metallothionein family such as MT1G (**Figure 2B**).

Enrichment analysis of 300 DEGs was performed using clusterProfiler and DAVID database. The enrichment results indicated that biological processes such as cellular response to copper ion and cellular response to zinc ion were significantly enriched, as shown in **Figure 2C** and **Supplementary Figure 2A**. This suggests that Fn is likely involved in the regulation of these biological processes to exert its effects. In the top five pathways identified through KEGG pathway enrichment analysis, both the clusterProfiler and DAVID databases highlighted the same pathways (**Figure 2D**, **Supplementary Figure 2B**), including mineral absorption, peroxisome proliferator-activated receptor (PPAR) signaling pathway, and apoptosis, among others.

### 3.2 *Fn* enhances the number of fibroblasts in the tumor microenvironment of AOM/DSS mice

**Figure 3:**
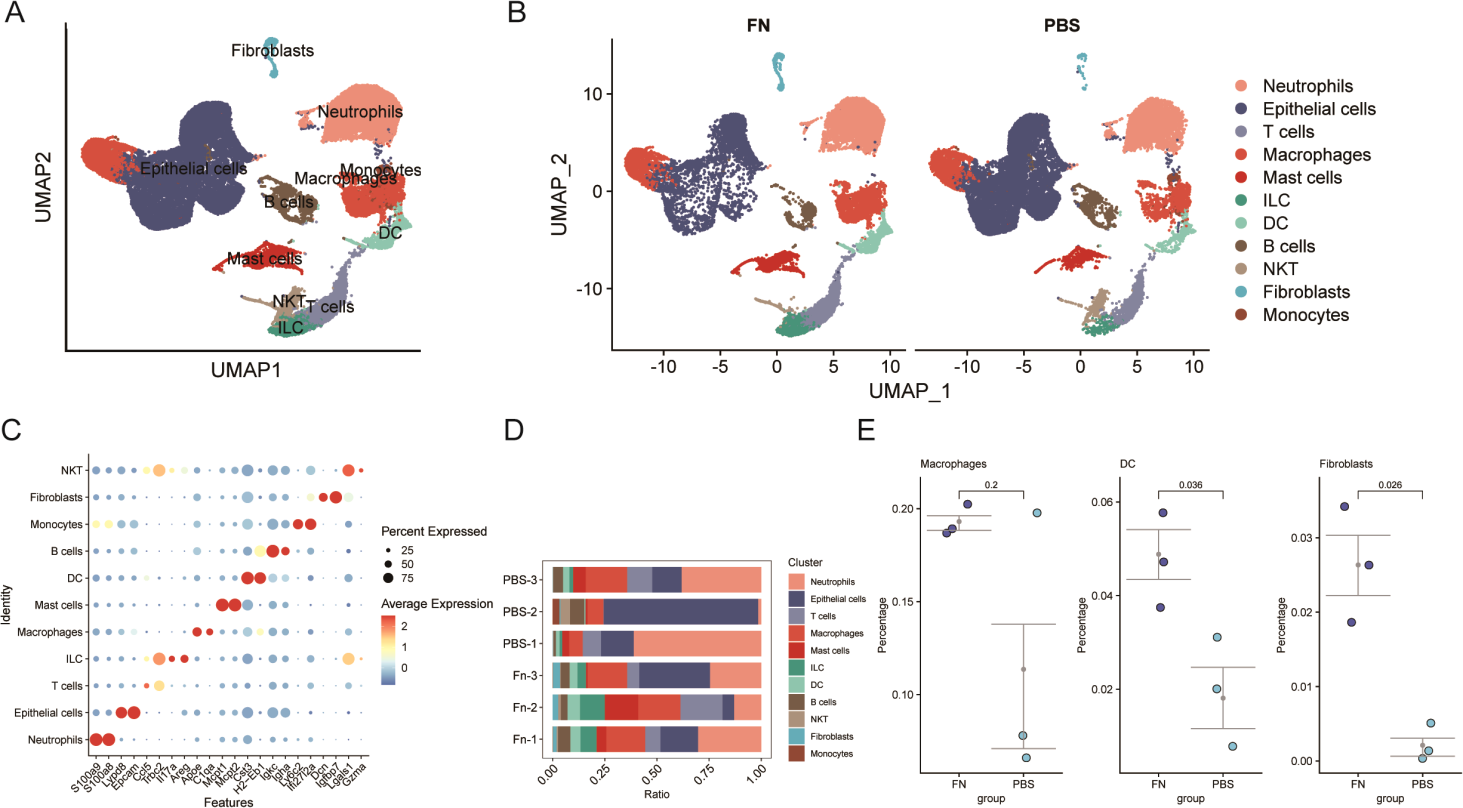
Single-cell transcriptome reveals a significant increase in the proportion of fibroblasts after *Fn* intervention (GSE172334). **(A, B)** Umap plots of cell clustering. **(C)** Maker genes of different cell clusters. **(D, E)** Comparison of cell proportions in different samples and between groups.

After eliminating the low expression genes and mitochondria-related genes (**Figure 3A**), the downscaled and clustered annotations were made into 11 cell types (**Figure 3A**). The cluster profile score was assessed using the silhouette function from the cluster package, yielding a value of 0.4675184, whereas the Calinski–Harabasz index was determined to be 3,909.427 using the calinhara function from the fpc package. These clustering metrics fall within an acceptable range, suggesting preliminary confirmation of clustering robustness. After further grouping into *Fn* vs. PBS groups, an increase in the number of fibroblasts was observed in the *Fn* group (**Figure 3B**). After demonstrating the cellular annotation of marker genes (**Figure 3C**), we quantified the proportions of various cell types within each sample (**Figure 3D**). A significant increase or decrease in the proportion of a specific cell type, which may indicate an alteration in its function, suggests that this cell type could be pivotal in the progression of *Fn*-induced colon cancer. Our findings did not reveal significant alterations in the proportion of epithelial cells; however, we observed notable changes in the proportion of fibroblasts. A statistically significant increase in the proportion of fibroblasts was indeed found in the *Fn* sample (**Figure 3E**). Additionally, we discovered that the *Fn* group had a considerably higher proportion of dendritic cells. There was increase in macrophages, but because there was a sample with a suspected abnormal increase in the PBS group, no statistically significant difference between the groups was observed. Here, we identify them as differentially proportioned cells.

### 3.3 *Fn* enhances fibroblast–macrophage–epithelial cell interactions in AOM/DSS mice

**Figure 4:**
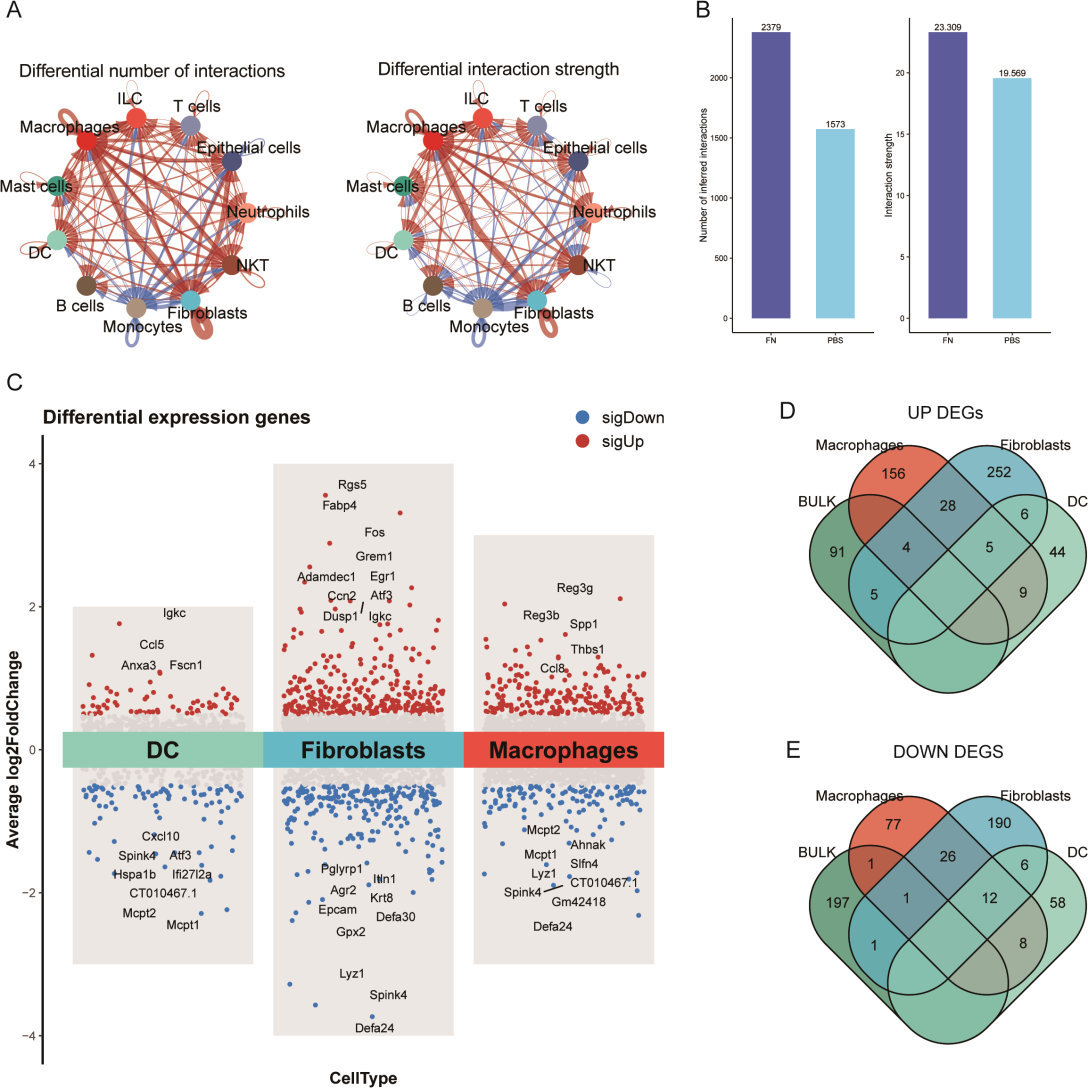
Identified differential genes between fibroblasts and dendritic cells (GSE172334). **(A)** The number of fibroblast–epithelial cell communication was significantly higher after *Fn* intervention. **(B)** The number and intensity of cell communication. **(C)** Volcano plots of fibroblast, macrophage, and dendritic cell differential analysis. **(D, E)** Extracted upregulated and downregulated differential genes to take the intersection, respectively.

In cellular communication, we noticed that the number and the intensity of fibroblasts interactions with themselves and macrophages and epithelial cells were significantly elevated (**Figure 4A**). Moreover, *Fn* increased the overall number and the intensity of interactions among cells in the tumor microenvironment (**Figure 4B**). To further observe which genes were altered by *Fn* in the differentially proportioned cells of the microenvironment, we extracted the corresponding cell clusters separately, screened the DEGs with the same thresholds (*P* < 0.05, |log2FC| > 0.5), and plotted the volcano plots (**Figure 4C**). Subsequently, we took the intersection of up/downregulated DEGs from differentially proportioned cells with up/downregulated DEGs from bulk RNA-seq, respectively (**Figures 4D, E**). Twelve candidate genes (9 upregulated and 3 downregulated) were finally obtained. Notably, no overlap of DEGs between dendritic cells and bulk RNA-seq was found.

### 3.4 Machine learning algorithm to identify feature genes

**Figure 5:**
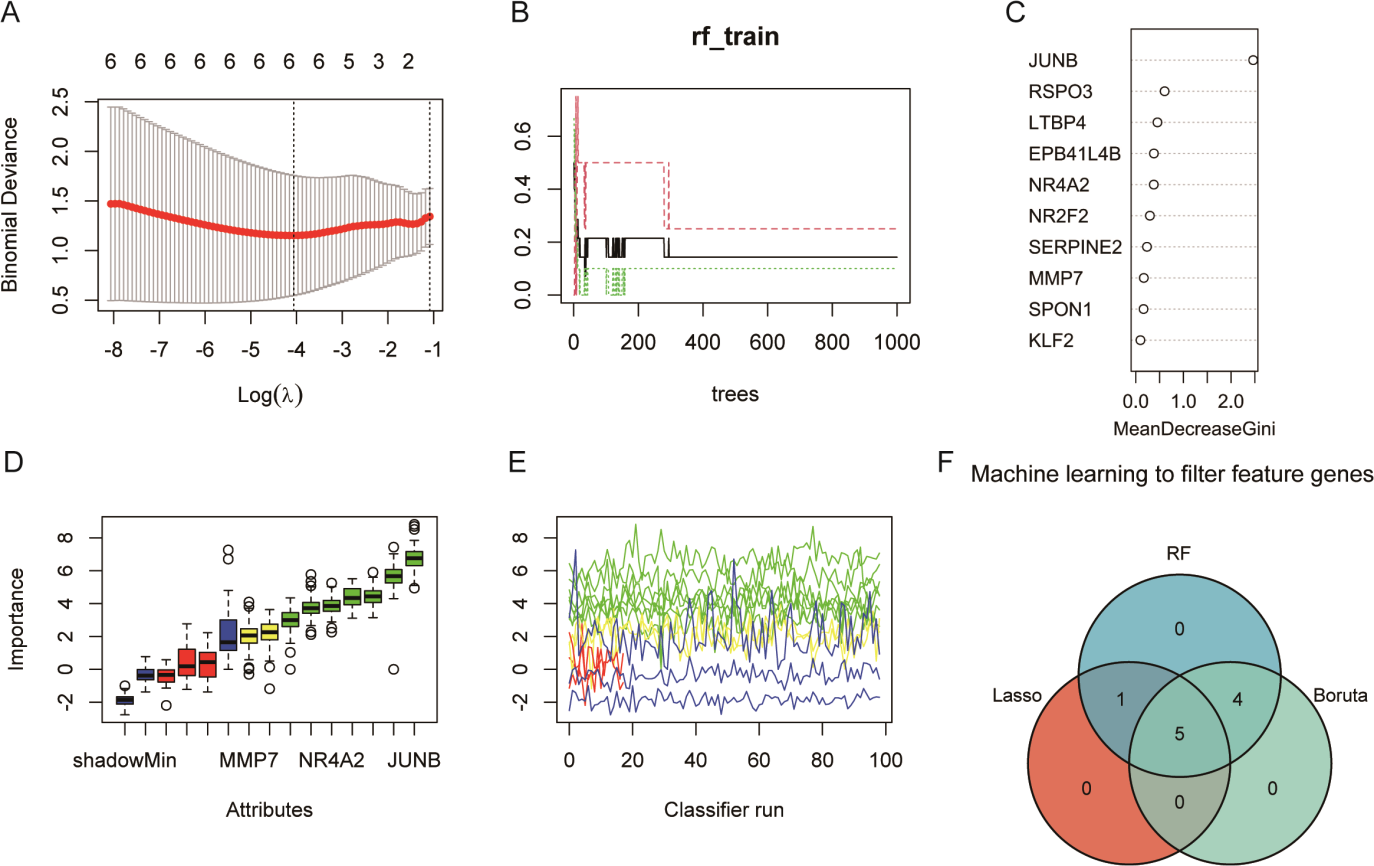
Machine learning identifies five feature genes (GSE122183): JUNB, SERPINE2, NR2F2, LTBP4, and MMP7. **(A)** Lasso regression. The panel shows a plot of the mean squared error in Lasso regression as a function of Log(λ), thus helping us to select the best model. The upper horizontal coordinate, showing the number of variables required for the corresponding model, decreases from left to right; the lower horizontal coordinate, the logarithm of the penalty coefficient λ; and the vertical coordinate indicates that the mean squared error (MSE) refers to the degree to which the predicted value of the computational model differs from the true value. **(B)** The model error rate remains stable after the random forest decision tree reaches 200. **(C)** Random forest screening of importance variables. **(D)** The blue box-and-line plot on the left of Boruta algorithm corresponds to the minimum, average, and maximum Z scores for a shadowed feature. **(E)** The green line corresponds to a confirmed feature, the red color indicates a rejected feature, the yellow color indicates a feature to be determined, and the blue color indicates the importance of a minimal, average, and maximal shadowed feature, respectively. **(F)** The three algorithms take the intersection to obtain the five key feature genes.

The lasso regression results were obtained six feature genes (**Figure 5A**). Random forest results suggest that the error rate starts to stabilize when the tree is greater than 400 (**Figure 5B**), so set the tree to 1,000 and take top 10 as the feature gene (**Figure 5C**). In Boruta algorithm, seven confirmed feature genes with two pending feature genes were obtained (**Figures 5D, E**). Five overlapping genes (Junb, Serpine2, Nr2f2, Ltbp4, and Mmp7) were obtained by plotting Venn graphs, and they were used as the feature genes identified by machine learning for the next analysis (**Figure 5F**).

### 3.5 Validation of feature genes

**Figure 6:**
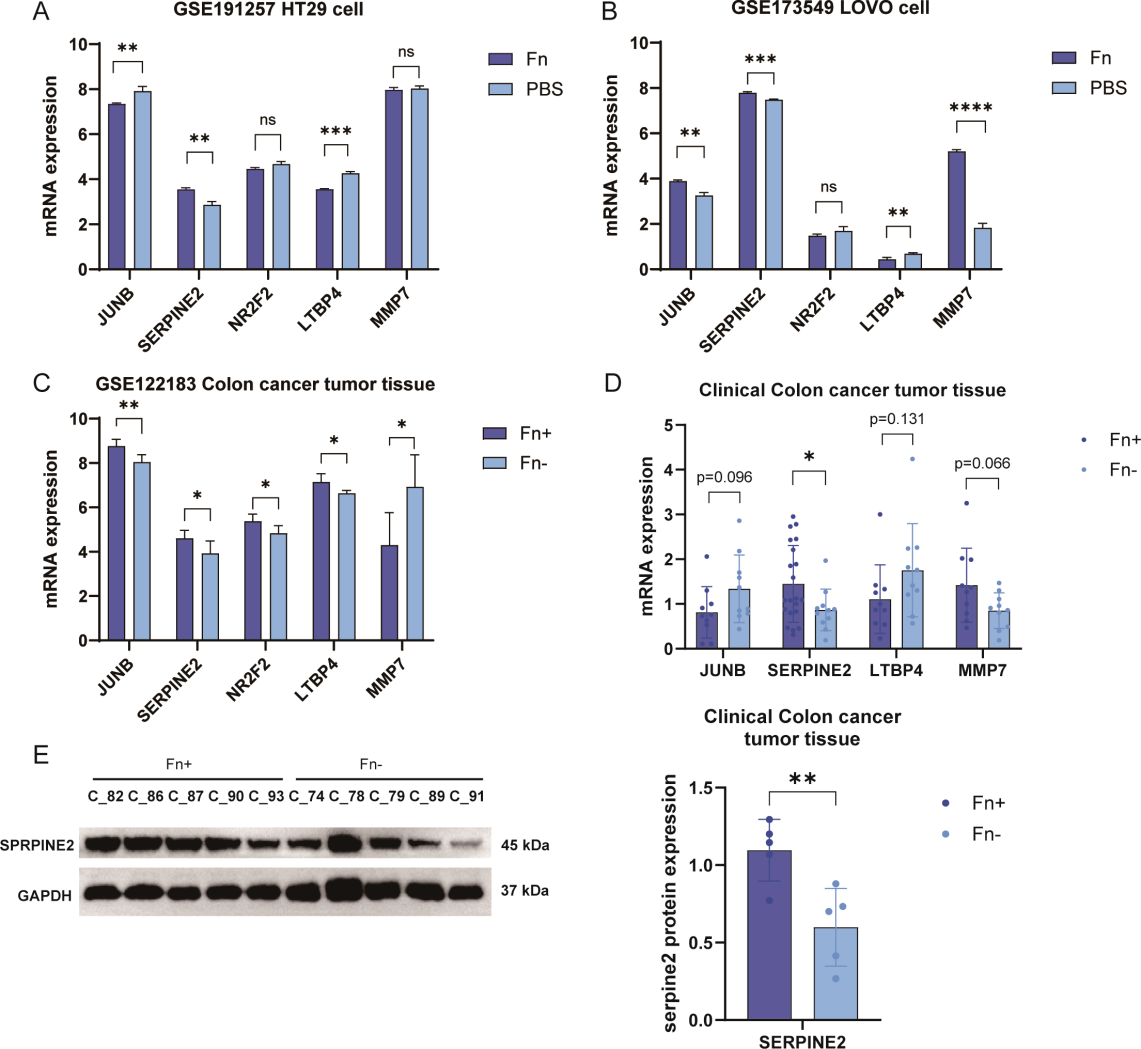
*Fn* co-culture significantly increased Serpine2 gene expression in different tumor cells. **(A, B)** Sequentially, the expression box plots of 5 featured genes were plotted using two external datasets (A, GSE173549, LoVo cells; B, GSE191257, HT29 cells). **(C)** Expression histograms of five featured genes were plotted using the training dataset. **(D)** The expression level of JUNB was detected in 60 clinical samples by using q-PCR, Serpine2, ltbp4, and mmp7E mRNA levels. **(E)** Western blotting was used to assess the expression levels of Serpine2 protein in 10 clinical samples. "*" means p < 0.05, "**" means p < 0.01, "***" means p < 0.001, and "****" means p < 0.0001.

In order to confirm that the differential genes that we identified are robust results triggered by *Fn*, we obtained two datasets of *Fn* co-cultured with human-derived colon cancer cell lines containing LoVo and HT29 (**Figures 6A, B**) in public databases. After retrieving and extracting the expression data of the five characterized genes, it turned out that only the Serpine2 gene showed an upward trend in different datasets, and the difference was significant in the two datasets. In contrast, although JUNB and LTBP4 also differed significantly in both datasets, the expression trends were not uniform with the analyzed dataset (**Figure 6C**). MMP7, on the other hand, was only validated in one external dataset with the opposite trend. Significant differences were not observed for NR2F2 in all validation datasets.

We used clinical samples for testing. We collected tumor pathology tissues from 32 patients with colon cancer at Sichuan Hospital of Traditional Chinese Medicine, and their clinical information is shown in **Supplementary Table 1**. The abundance of *Fn* in the tumor tissues of different patients was obtained by 5 Region 16S rRNA gene sequencing (5R) analysis, and this was grouped into *Fn*+ vs. *Fn*−. PCR results suggested that the tumor tissues of patients with *Fn*+ had significantly increased mRNA levels of the Serpine2 gene (**Figure 6D**), and no significant difference was observed for the other three genes no significant difference was observed. Western blotting suggested that the protein level of Serpine2 gene was increased in tumor tissues of *Fn*+ patients (**Figure 6E**). We finally retained only the Serpine2 gene as the key gene.

### 3.6 Serpine2 expression is significantly associated with pathological stage/tumor metastasis in colon cancer

**Figure 7:**
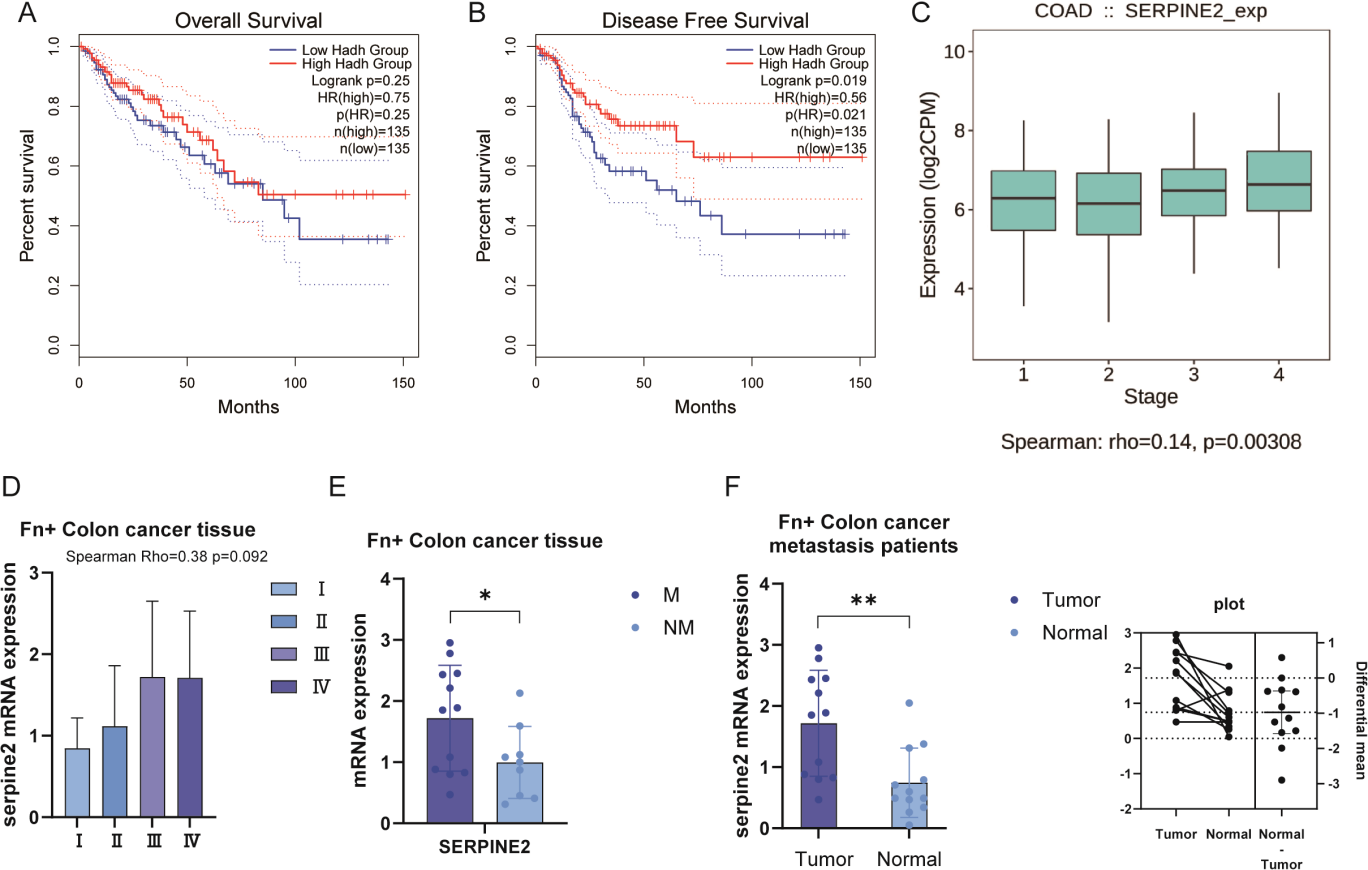
Serpine2 was positively correlated with pathological stage of colon cancer. **(A, B)** In the TCGA dataset, Serpine2 was not observed to be correlated with OS and DFS outcomes of patients. **(C)** The TCGA dataset showed a positive correlation between Serpine2 mRNA expression and pathological stage. **(D)** In our clinical samples, correlation was observed between Serpine2 mRNA expression and pathological stage (*p* = 0.092). **(E)** In *Fn*+ colon cancer tumor tissues, Serpine2 mRNA expression was higher in metastatic samples (M) than in non-metastatic samples (NM). **(F)** In *Fn*+ metastatic colon cancer patients, Serpine2 mRNA expression was higher in tumor samples than in paired normal tissue (paired t-test). "*" means p < 0.05 and "**" means p < 0.01.

We collected The Cancer Genome Atlas (TCGA) data for functional analysis of Serpine2. In the KM curve, Serpine2 was not observed to be a significant risk prognostic gene (**Figures 7A, B**). However, we found that Serpine2 mRNA expression level was significantly correlated with the pathological stage of colon cancer, and Serpine2 gene expression level with deeper stage (**Figure 7C**).

The results of our collected clinical samples also reconfirmed the correlation between Serpine2 mRNA expression level and pathological stage (**Figure 7D**), although *p* = 0.092. Further subgroup analysis suggested that Serpine2 mRNA expression was higher in metastatic samples than in non-metastatic samples in *Fn*+ colon cancer tumor tissues (**Figure 7E**). In addition, we also analyzed the tumor tissues of *Fn*+ metastatic colon cancer patients with normal tissues for comparative assays. Patients with *Fn*+ metastatic had higher levels of Serpine2 mRNA expression in their tumor samples compared to matching normal tissues (**Figure 7F**).

### 3.7 Localization and regulatory network analysis of Serpine2

**Figure 8:**
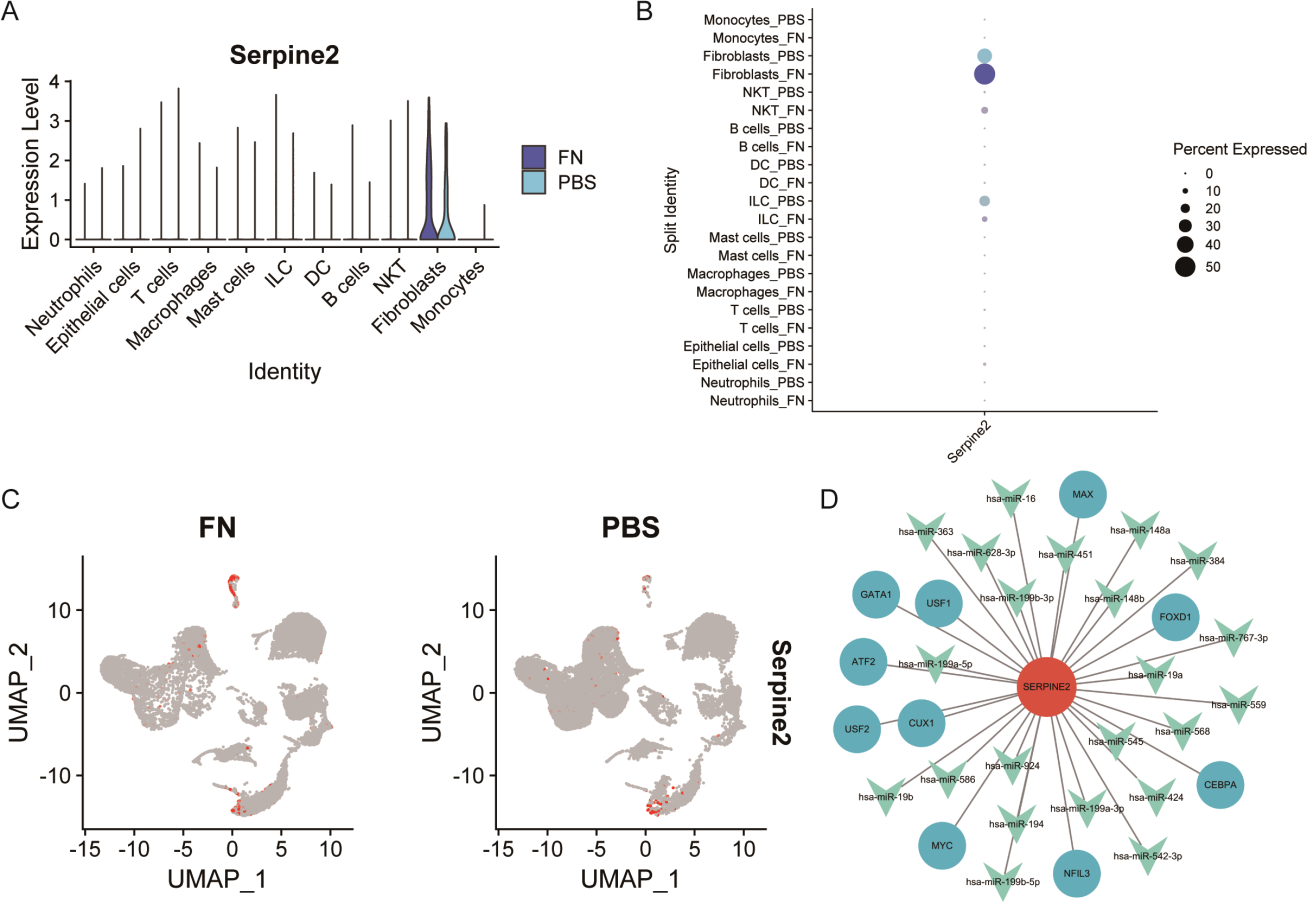
Analysis of Serpine2 localization and regulatory network. **(A–C)** Single-cell analysis suggested that Serpine2 was mainly localized on fibroblasts and the Serpine2 mRNA expression level was higher in the *Fn* group than that in the PBS group (GSE172334). **(D)** Constructed transcription factor–miRNA–gene regulatory network.

After identifying Serpine2 as a key gene, we wanted to further observe its localization and function. By scRNA-seq, we found that Serpine2 was mainly distributed on fibroblasts and was higher in the *Fn* group than in the PBS group (**Figure 8A**). We also noted that Serpine2 was also distributed on intrinsic lymphocytes, i.e., Innate Lymphoid Cells (ILCs) (**Figures 8B, C**). Finally, a TF-gene-miRNA regulatory network was constructed based on Serpine2, predicting 10 transcription factors with 27 miRNAs (**Figure 8D**).

### 3.8 *Fn* co-cultures with fibroblasts markedly enhance tumor cell growth and migration

**Figure 9:**
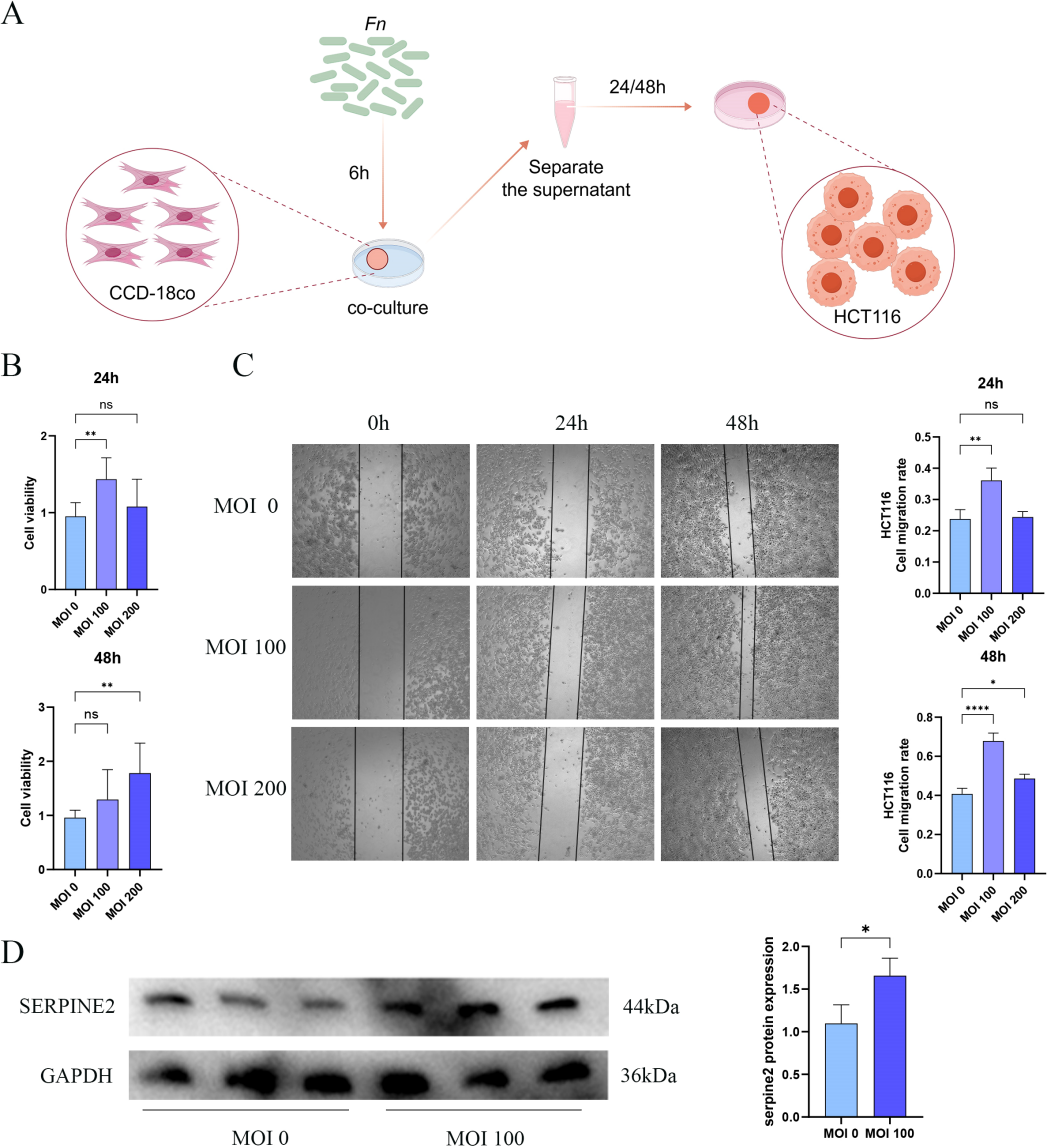
The supernatant derived from the co-culture of *Fn* and CCD-18Co cells significantly promotes the growth and migratory capabilities of tumor cells. **(A)** The schematic representation of the co-culture setup involving *Fn* and CCD-18Co cells. **(B)** The CCK-8 assay to evaluate the impact of the *Fn*-fibroblast co-culture supernatant on the proliferation of HCT116 cells, where MOI of 0 indicates the use of supernatant from fibroblasts cultured with uninfected *Fn*, whereas MOIs of 100 and 200 correspond to fibroblasts cultured with varying levels of *Fn* infection. **(C)** The scratch assay to assess the influence of the *Fn*-fibroblast co-culture supernatant on the migratory capacity of HCT116 cells. **(D)** The Western blot analysis, indicating a significant increase in the protein levels of Serpine2 in fibroblasts following *Fn* infection. Data are expressed as the mean ± standard deviation (SD), and statistical significance was evaluated using one-way ANOVA, with results denoted as *p < 0.05, **p < 0.01 and ****p < 0.0001. ns, Not Significant.

We further corroborated the findings from the aforementioned analyses and examined the function of Serpine2 through co-culture experiments involving fibroblasts and *Fn* (**Figure 9A**). The CCK-8 assay indicated that the supernatant from co-cultures with *Fn* at MOIs of 100 and 200 demonstrated a pronounced pro-proliferative effect on the HCT116 colon carcinoma cell line (**Figure 9B**). Notably, in the group at MOI of 100, the relative viability of HCT116 cells was significantly elevated at 24 h compared to the group at MOI of 0. Subsequently, we evaluated cell migration using a scratch assay, which revealed that the *Fn*-fibroblast co-culture supernatant significantly expedited the wound closure process. The most pronounced scratch healing rate was observed in the group at MOI of 100 at both 24 h and 48 h (**Figure 9C**). This pro-migratory effect is strongly associated with the increased tumor metastasis observed clinically in *Fn*-positive colon cancer patients. Based on the results from the CCK-8 and scratch assays, we selected fibroblasts from MOI of 0 and MOI of 100 for further molecular-level validation. The findings indicated that the protein expression level of Serpine2 was significantly elevated in fibroblasts co-cultured with *Fn* compared to those cultured without *Fn* (**Figure 9D**).

## 4 Discussion

Colon cancer ranks among the leading causes of cancer-related mortality globally. Recent advancements in technologies, such as multi-omics, have increasingly demonstrated the pivotal role of microorganisms in the progression of colon cancer. Notably, *Fn*, an oral-origin microorganism, has been implicated as a causative agent in colon cancer. Contemporary foundational studies have highlighted the involvement of *Fn* in colon cancer progression, metastasis, and the molecular mechanisms underlying immunotherapy (8–10, 18–20). Nevertheless, the precise mechanisms by which *Fn* contributes to colon cancer development remain inadequately understood. Consequently, elucidating the oncogenic role of *Fn* and its associated mechanisms is crucial for the identification of novel personalized diagnostic and therapeutic strategies for colorectal cancer. To comprehensively investigate the molecular mechanisms by which *Fn* promotes colon cancer progression, we have designed the present study.

Initially, we conducted a differential analysis of the collected bulk RNA sequencing data, identifying 300 genes that were differentially expressed in *Fn*-positive colon cancer tumor tissues. These DEGs were predominantly associated with GO annotations related to metal ions. It is well established that the tumor microenvironment comprises a complex assembly of epithelial cells, extracellular matrix, and various non-epithelial cell types, such as fibroblasts. Within this intricate microenvironment, numerous interactions among these components can precipitate tumor proliferation and metastasis (21). However, differential gene expression analysis based solely on bulk RNA-seq may not adequately elucidate the effects of *Fn* on the colon cancer tumor microenvironment. scRNA-seq enables the examination of differential gene expression at the resolution of individual cells. For instance, König et al. (22) demonstrated, through scRNA-seq, an increase in specific macrophage subtypes, ultimately confirming that the knockdown of Mir34a in bone marrow cells could inhibit the polarization of tumor-associated macrophage M2, thereby delaying the progression of colon cancer. To further elucidate the alterations within the tumor microenvironment, we employed single-cell sequencing analysis. Our findings indicate that intervention with *Fn* led to an increased ratio of fibroblasts, macrophages, and dendritic cells.

These cellular populations have been consistently reported to be closely associated with the progression of colon cancer (23–25). For instance, the gene expression profile of cancer-associated fibroblasts (CAFs) has demonstrated significant prognostic value in patients with colon cancer (26). Additionally, inhibiting the polarization of macrophages can remodel the immunosuppressive microenvironment, thereby enhancing the efficacy of antiangiogenic therapy in colon cancer (27). Excessive activation of dendritic cells has been shown to promote colon tumorigenesis in murine models (28). Concurrently, the gut microbiota can influence the recruitment, activation, and function of these cellular populations within the tumor microenvironment, thereby shaping the immunosuppressive and pro-tumorigenic ecological niche characteristic of colon cancer (29). *Fn* has been implicated in the regulation of these cellular populations; for example, it can promote macrophage M1 polarization through Tumor Necrosis Factor Superfamily Member 9 (TNFSF9)/Interleukin-1 Beta (IL-1β) signaling pathways (30). *Fn* has been shown to significantly enhance the activation of fibroblast activation proteins, which are implicated in the invasive behaviors of CAFs in colorectal cancer (31). This observation suggests that *Fn* may activate or recruit these cell populations within the tumor microenvironment of colon cancer, thereby facilitating cancer progression.

To evaluate the extent of cellular interactions, we conducted a cellular communication analysis. Our findings indicate that *Fn* increases both the number and strength of cellular interactions, particularly between fibroblasts and macrophage/epithelial cells. The interaction between CAFs and the tumor immune microenvironment is recognized as a critical factor in tumor progression (32). CAFs are activated through the secretion of various cytokines (33), chemokines (34), and other effector molecules. These molecules interact with tumor-infiltrating immune cells within the tumor microenvironment, contributing to the formation of an immunosuppressive environment that allows cancer cells to evade immune surveillance. Research has demonstrated that co-culturing macrophages, CAFs, and tumor cells enhances the invasive potential of colon tumor cells. This process is linked to the role of CAFs in recruiting and facilitating the differentiation of infiltrating monocytes into specific macrophage populations (35). Notably, there is a significant increase in the proportion of fibroblasts, leading us to hypothesize that enhanced interactions between fibroblasts and macrophages are critical mechanisms through which *Fn* promotes the progression of colon cancer.

Subsequently, we aimed to identify key genes that are differentially expressed both at the tissue level and within the aforementioned cell types. These genes are promising candidates for early predictive diagnostics and are suitable as molecular markers for mechanistic studies at the cellular level. To achieve this, we examined the intersection of DEGs in these cells with those identified in bulk RNA sequencing of tissues. Our analysis revealed that the majority of overlapping genes were derived from fibroblasts, a smaller number from macrophages, and none from dendritic cells. This supports our previous discussion that the increase in the fibroblast-macrophage ratio and the strengthening of interactions that promote colon cancer progression may be attributed to *Fn*, with the overlapping genes predominantly originating from fibroblasts and macrophages. After identifying 12 co-DEGs, we utilized three machine learning algorithms to screen and characterize five specific genes, ensuring the robustness and reliability of our findings rather than attributing them to chance. For validation, we employed sequencing data from two additional human-derived colon cancer cell lines co-cultured with *Fn*. Ultimately, we identified Serpine2 as the key gene, which was significantly upregulated in *Fn*-positive tissues or colon cancer cells.

Serpine2, also known as protease nexin–1, primarily influences the metabolism of extracellular matrix proteins by inhibiting the activity of related proteases within the extracellular matrix (36). As early as 2003, it was discovered that the overexpression of Serpine2 altered intra-tumor Extracellular Matrix (ECM) production and enhanced the invasive potential of pancreatic cancer cells in xenograft models using nude mice (37); whereas, in other tumors, Serpine2 has been shown to promote lung cancer (38), hepatocellular carcinoma (39), breast cancer (40), esophageal squamous cell carcinoma (41), melanoma (42), prostate cancer (43), and oral squamous cell carcinoma (44) progression or metastasis. In the domain of colon cancer research, several studies have identified a significant inverse correlation between Serpine2 expression and microsatellite instability scores (45). Additionally, Serpine2 expression is markedly upregulated in adenomas with Apc mutations (46). Our analysis of TCGA dataset corroborates these findings, revealing a significant association between Serpine2 expression and pathological staging. This aligns with previous research indicating that Serpine2 expression is notably elevated in advanced colon adenocarcinoma tumors (14). These findings suggest a potential link between Serpine2 and specific clinical subtypes of colon cancer. Furthermore, recent studies have explored the mechanisms underlying colon cancer progression (14, 46). Notably, Serpine2 is upregulated in response to oncogenic activation of Rat Sarcoma viral oncogene homolog (Ras), B-Raf proto-oncogene, serine/threonine kinase (BRAF), and Mitogen-activated protein kinase kinase 1 (MEK1), contributing to the pro-tumorigenic effects of ERK signaling in intestinal epithelial cells (47). In alignment with stroma-associated colon cancer markers, such as MMPs, Serpine2 is predominantly secreted by stromal cells and plays a functional role in this context. However, unlike MMPs, which are primarily implicated in extracellular matrix remodeling and have established roles in epithelial proliferation, ECM homeostasis, angiogenesis, and inflammatory responses, as well as being promising targets for therapeutic intervention in colon cancer (48), research on Serpine2 in tumorigenesis remains nascent. This category of stromal cell-secreted pro-cancer markers holds significant potential, either as indicators of disease progression or as novel therapeutic targets, offering promising avenues for targeted therapy and prognostic evaluation. These investigations offer insights into how *Fn* may facilitate colon cancer progression through the enhancement of Serpine2 expression.

We collected clinical samples from patients with colon cancer and reaffirmed the potential of Serpine2 as a molecular marker for *Fn*-promoted colon cancer progression. The expression levels of Serpine2, both at the mRNA and protein levels, were elevated in *Fn*-positive colon cancer tumor tissues compared to those in *Fn*-negative tissues. Furthermore, Serpine2 expression was correlated with the pathological stage of the disease (Rho>0.3), although the p-value is not significant (*p* = 0.092). Subgroup analysis indicated that the mRNA levels of Serpine2 were notably higher in metastatic patients than in non-metastatic patients, specifically within *Fn*-positive tumor tissues. Additionally, the mRNA levels of Serpine2 in tumor tissues from metastatic patients were significantly greater than those in paired normal colon tissue samples. In fact, Serpine2 can contribute to tumor metastasis by enhancing the permeability of extravascular matrix and promoting the formation of vascular-like network structures in epithelial cells was reported in *Nature* (49). This suggests that Serpine2 may possess pro-metastatic potential in colon cancer.

Importantly, our findings revealed that Serpine2 was predominantly localized to fibroblasts. Recent research has demonstrated the pro-cancer potential of Serpine2 when functioning from an exosome. They revealed that the expression of Serpine2 in exosomes secreted by CAFs was significantly elevated compared to those secreted by normal fibroblasts, thereby enhancing the malignant phenotype of lung cancer cells. The overexpression of Serpine2 in CAF-derived exosomes was found to further augment the malignant phenotype of lung cancer cells (38). These findings provide some evidence for our hypothesis regarding the role of Serpine2 in promoting colon cancer metastasis through the exosome pathway. Based on the results of cellular communication studies, which indicated an elevated level of fibroblast–epithelial cell interactions, we hypothesize that a similar mechanism may be present in colon cancer. Specifically, *Fn* may be appears to stimulate fibroblasts to release exosomes enriched with Serpine2, which subsequently act on colon cancer cells to enhance their metastatic potential. To explore this hypothesis, we constructed a regulatory network centered around Serpine2, identifying potential transcription factors and miRNAs involved in its regulation. A substantial number of these regulatory molecules have been previously implicated in colon cancer metastasis. For instance, the proto-oncogene MYC has been associated with liver metastases in colon cancer (50). Likewise, it has been demonstrated that inhibiting Forkhead box protein D1 (FOXD1), another well-known oncogene, decreases colon cancer cells’ capacity for invasion and migration (51). Additionally, Activating transcription factor 2 (ATF2) is known to participate in colorectal cancer by negatively regulating the transcription of miR-3913-5p through promoter binding (52). While these regulatory molecules have been predicted, further investigation is required to elucidate their role in modulating Serpine2 and promoting colon cancer metastasis. In future research, the upstream mechanisms of Serpine2 in colon cancer metastasis should be further explored through experimental screening and validation.

To substantiate our findings, we conducted a co-culture of *Fn* with CCD-18co colon fibroblasts and subsequently collected both the *Fn*-infected CCD-18co cells and the co-culture supernatants. Upon utilizing the co-culture supernatant to culture HCT116 colon cancer cells, we observed a marked enhancement in the proliferative and migratory capacities of the HCT116 cells. This observation further corroborates the hypothesis that *Fn* can influence fibroblasts, thereby facilitating the progression of the malignant phenotype in colon cancer cells. Additionally, we assessed the levels of Serpine2 protein in CCD-18co cells, finding a significant upregulation in *Fn*-infected cells. These results further support the role of *Fn* in promoting colon cancer progression and metastasis through the upregulation of Serpine2 expression in fibroblasts. However, this study did not provide a detailed classification of fibroblasts and lacked an exploration of CAFs heterogeneity. Further subdivision into fibroblast subpopulations with distinct characteristics could facilitate the tracing of Serpine2’s origin and elucidate deeper mechanisms. Recent research has demonstrated that, in the colorectal cancer microenvironment, fibroblasts can be categorized into four clusters: myofibroblast CAF–like, inflammatory CAF–like, proliferative fibroblasts, and a newly identified cluster termed “T-cell inhibitory CAF”. This finding underscores the influence of a novel class of immunosuppressive CAFs on colon cancer within the tumor microenvironment (53). Furthermore, although the findings have been validated through *in vitro* co-culture experiments using clinical samples, caution is advised when applying these results in future studies due to the inherent differences between humans and mice.

Although this study was validated by multiple public datasets and our clinical cohort, which initially confirmed the robustness of the findings, the following limitations remain for further improvement: first, the total sample size of the training set included in the analysis was relatively small and the sample type was limited to tumor tissues. In the future, there is a need to expand the sample size and collect blood samples, which will significantly improve the reliability and clinical translational potential of the study results. Secondly, single-cell sequencing data from animal models suggested that *Fn* infection promotes colon cancer progression through upregulation of Serpine2, which we further verified by RNA-seq of *Fn* co-cultured with HT29 and CT26 cells, respectively. The shortcomings are that some details of *Fn* infection and colonization are difficult to complete due to the missing information in the public dataset, and the transcriptome validation data at the tissue level are still insufficient. To enhance the precision of our findings, we recruited patients diagnosed with colon cancer and obtained samples of their colon tumors along with distal normal tissues. These patients were categorized into *Fn*-positive and *Fn*-negative groups using 16S Ribosomal RNA (rRNA) detection. Notably, in comparison to *Fn*-negative patients, there was a significant upregulation of Serpine2 mRNA and protein expression in *Fn*-positive tumor samples. Furthermore, we identified a significant correlation between Serpine2 expression levels and the progression of colon cancer, as determined by the pathological stage of the patients. Given that Serpine2 has been implicated in the metastasis of other cancer types, we also gathered clinical data regarding colon cancer metastasis during patient recruitment. Through subgroup analyses, we confirmed a strong association between Serpine2 expression and colon cancer metastasis. Due to ethical considerations related to human subjects, an interventional study design for *Fn* was not feasible, thereby complicating the exclusion of potential confounding factors. Moreover, the mechanisms by which *Fn* regulates Serpine2 and influences the tumor microenvironment remain insufficiently explored. In future research, the integration of a CRISPR-Cas9–mediated fibroblast Serpine2 knockdown model with a three-dimensional organoid co-culture system could facilitate a systematic evaluation of the impact of Serpine2-enhanced Fibroblast-epithelial cell interactions on the malignant characteristics of tumors, such as invasion, migration, and proliferation. Such comprehensive studies are anticipated to elucidate the molecular mechanisms through which *Fn* augments Serpine2-driven fibroblast–epithelial cell interactions, thereby laying the groundwork for potential clinical applications.

## 5 Conclusion

In conclusion, our study demonstrates that *Fn* facilitates the progression of colon cancer by augmenting interactions between fibro-macrophages and epithelial cells. Concurrently, we have identified Serpine2 as a potential molecular marker associated with *Fn*-promoted colon cancer progression, with its expression predominantly localized to fibroblasts. Furthermore, the pro-metastatic potential of Serpine2 has been validated through clinical samples. Collectively, these mechanisms and biomarkers offer novel insights for future mechanistic investigations and hold promise for advancing drug discovery efforts aimed at treating this condition.

## Data Availability

The original contributions presented in the study are included in the article/**Supplementary Material**. Further inquiries can be directed to the corresponding author.
