# Supplementary figures and images for: Enhancing fibroblast–epithelial cell communications: Serpine2 as a key molecule in Fusobacterium nucleatum–promoted colon cancer
Source: Front Immunol. 2025 Jun 26;16:1563922. doi: 10.3389/fimmu.2025.1563922 (PMC12240788; doi:10.3389/fimmu.2025.1563922)

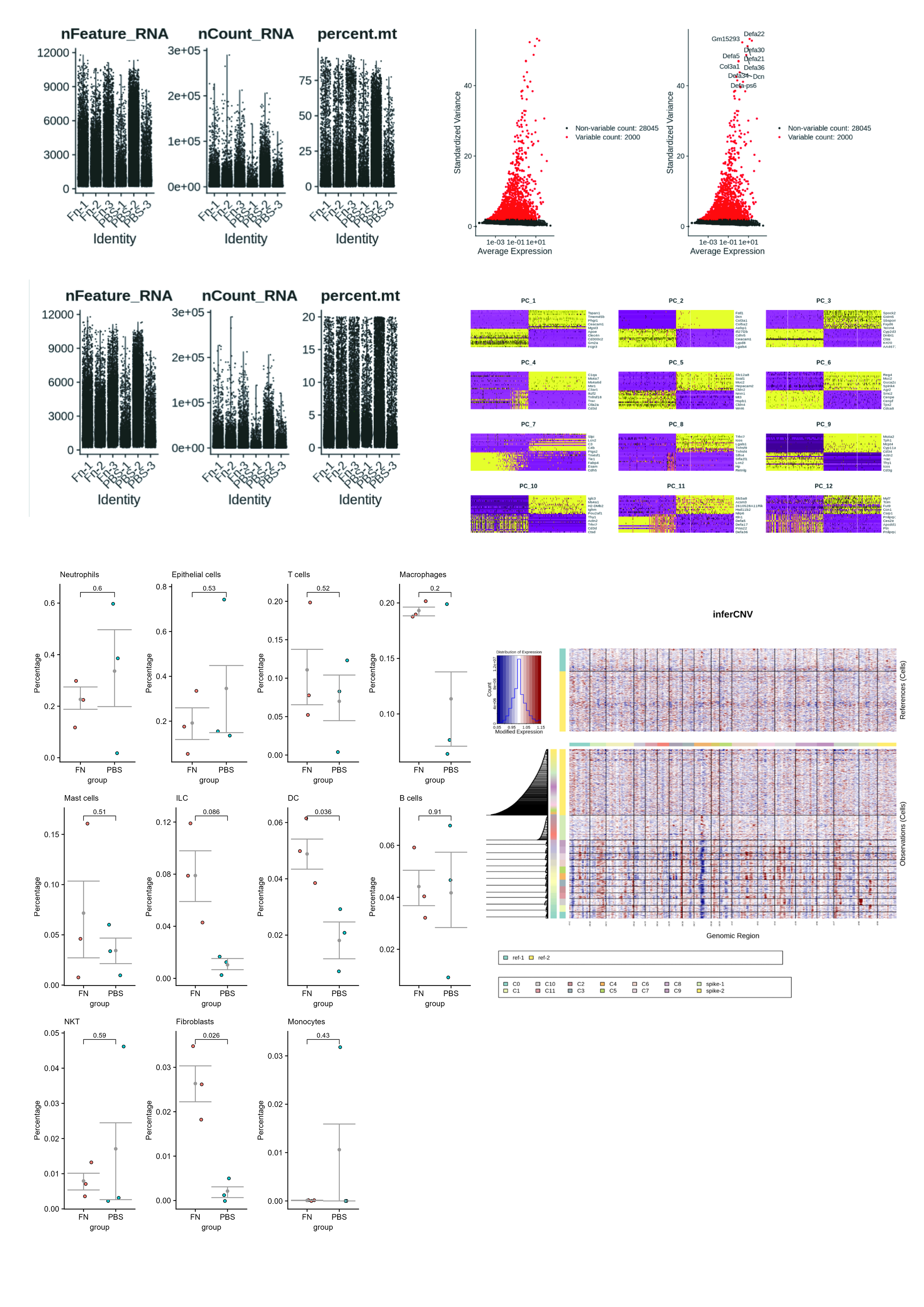

Supplement: Supplementary file 1 [file Image1.tif]

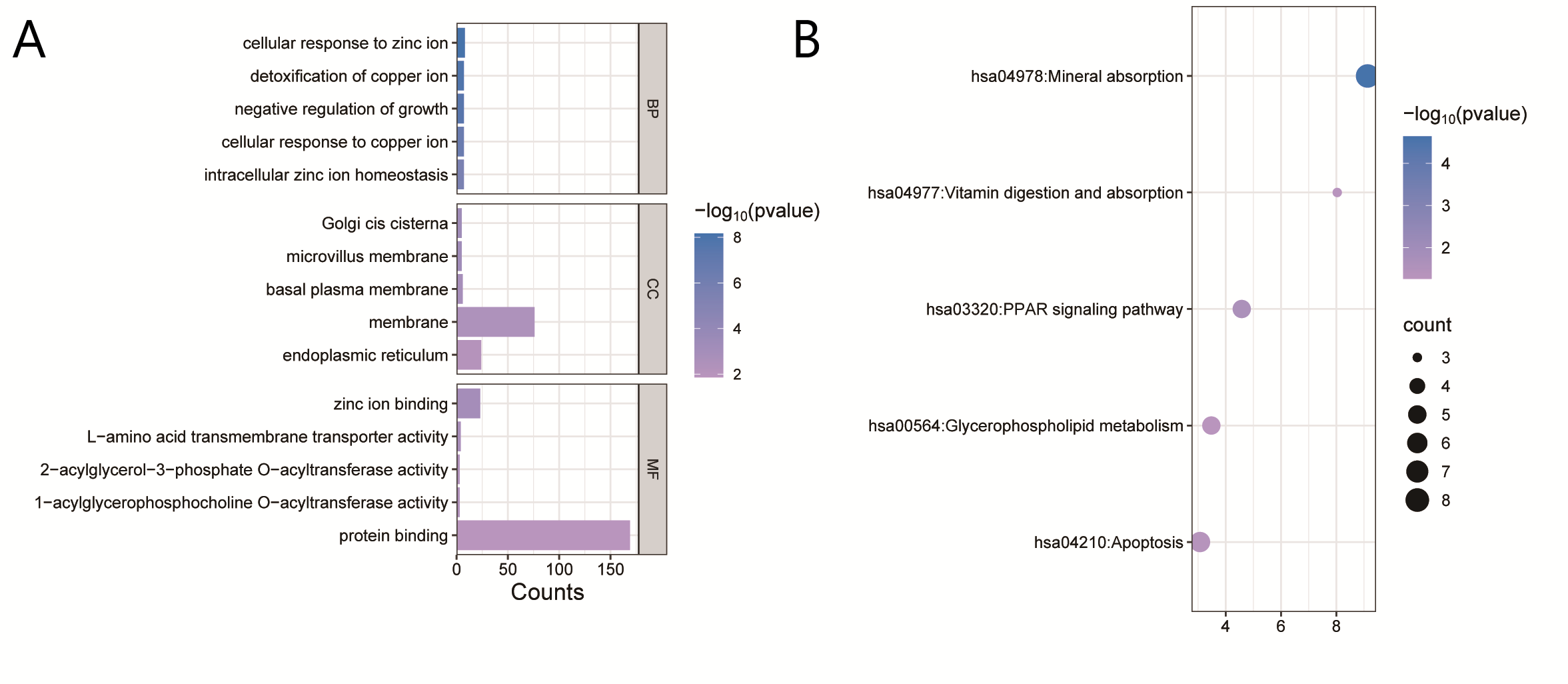

Supplement: Supplementary file 2 [file Image2.tif]
