# Supplementary material for: Enhancing fibroblast–epithelial cell communications: Serpine2 as a key molecule in Fusobacterium nucleatum–promoted colon cancer
Source: Front Immunol. 2025 Jun 26;16:1563922. doi: 10.3389/fimmu.2025.1563922 (PMC12240788; doi:10.3389/fimmu.2025.1563922)
Supplement: Supplementary file 3 [file Table1.docx]

**Table S1:** Demographics and Clinical Characteristics of the participants

|  |  | *Fusobacterium nucleatum* | |  |
| --- | --- | --- | --- | --- |
| Characteristics | Overall, N = 32*^1^* | Positive,  N = 21 (66%)*^1^* | Negative,  N = 11 (34%)*^1^* | *P* value*^2^* |
| Gender |  |  |  | 0.465 |
| Male (n, %) | 20 (62.50%) | 12 (57.14%) | 8 (72.73%) |  |
| Female (n, %) | 12 (37.50%) | 9 (42.86%) | 3 (27.27%) |  |
| Age (years) | 63.16 (9.82) | 63.00 (10.78) | 63.45 (8.17) | 0.895 |
| BMI (kg/m^2^) | 22.95 (3.00) | 23.00 (2.46) | 22.85 (3.99) | 0.916 |
| Smoking |  |  |  | 0.213 |
| Yes | 9 (28.13%) | 4 (19.05%) | 5 (45.45%) |  |
| No | 23 (71.88%) | 17 (80.95%) | 6 (54.55%) |  |
| Alcohol |  |  |  | 0.397 |
| Yes | 8 (25.00%) | 4 (19.05%) | 4 (36.36%) |  |
| No | 24 (75.00%) | 17 (80.95%) | 7 (63.64%) |  |
| TNM stage |  |  |  | 0.285 |
| I | 5 (15.63%) | 4 (19.05%) | 1 (9.09%) |  |
| II | 10 (31.25%) | 5 (23.81%) | 5 (45.45%) |  |
| III | 14 (43.75%) | 11 (52.38%) | 3 (27.27%) |  |
| IV | 3 (9.38%) | 1 (4.76%) | 2 (18.18%) |  |
| Tumor metastasis |  |  |  | 0.798 |
| Yes | 17 (53.13%) | 12 (57.14%) | 5 (45.45%) |  |
| No | 15 (46.88%) | 9 (42.86%) | 6 (54.55%) |  |
| *^1^* Mean (SD); n (%)  *^2^* Welch Two Sample t-test; Pearson’s Chi-squared test; Fisher’s exact test  Abbreviations: BMI, body mass index; TNM stage, tumor/lymph node metastasis/distal metastasis stage. | | | | |
